# Exploring quality standards implementation at a South African municipality’s health facilities

**DOI:** 10.4102/curationis.v46i1.2416

**Published:** 2023-06-21

**Authors:** Seani R. Matahela, Ayobami P. Adekola, Azwihangwisi H. Mavhandu-Mudzusi

**Affiliations:** 1 Department of Health Studies, College of Human Sciences, University of South Africa, Pretoria, South Africa; 2 Institute for Gender Studies, College of Human Sciences, University of South Africa, Pretoria, South Africa

**Keywords:** experience, health facilities, quality assurance, quality assurance manager, quality standards, compliance, healthcare, South Africa

## Abstract

**Background:**

Despite government initiatives to ensure the delivery of safe and high-quality care in health establishments, most health establishments in the City of Tshwane Metropolitan Municipality, South Africa were non-compliant with the National Core Standards. This study explored the experiences of quality assurance managers regarding quality standards implementation in these establishments.

**Objectives:**

This study aimed to explore and describe factors affecting the implementation of quality standards at public health facilities based on quality assurance managers’ lived experiences in the research setting.

**Method:**

This qualitative study used phenomenological design by conducting individual in-depth interviews with nine purposively selected quality assurance managers in 2021. The collected data were analysed using Colaizzi’s phenomenological analysis framework.

**Results:**

The study’s findings revealed that the legislative framework and the policy environment were motivators for quality standard compliance among the participants. Furthermore, human resources, materials-related issues and poor infrastructure were found to be barriers to the implementation of quality standards in health facilities.

**Conclusion:**

The explored and described barriers must be addressed to improve compliance with the National Core Standards at public health facilities in the City of Tshwane Metropolitan Municipality. In addition, capacity-building for quality assurance managers should be ongoing to ensure the highest quality implementation standards and to strengthen the enforcement of quality standard regulations.

**Contribution:**

The study’s findings explored and described the factors that influence the implementation of quality standards. Addressing these factors could improve the quality of healthcare delivery in the research setting’s health facilities.

## Introduction

Quality assurance in the health system is vital for ensuring that the appropriate quality standards are followed (Ojo, Tolentino & Yoon 2021:5). South Africa implemented a quality assurance system to assure quality healthcare delivery in public health institutions, by means of establishing operational norms and standards for the country’s health establishments (National Department of Health [NDoH] 2013:8). Various studies have shown that a well-implemented quality assurance system should deliver sustained and high-quality universal healthcare services (Friebel et al. 2018:4; Serrate 2019:1). The major barriers to the maintenance of quality assurance in African health establishments are poor governance, the shortage of human resources and inadequate policies and operating procedures (Kakyo & Xiao 2017:248). In South Africa, the Office of Health Standards Compliance (OHSC) developed the National Core Standards, which have been put in place to regulate the expected level of service delivery and operational guidelines for managers at all levels (NDoH 2013:23). Poor management and leadership, according to Maphumulo and Bhengu (2019:4), are barriers to maintaining quality standards in South African healthcare establishments. While the government has appointed quality assurance managers at the facilities concerned, so as to ensure compliance with the standards, most health establishments in the City of Tshwane Metropolitan Municipality district do not comply with the National Core Standards (OHSC 2017:54). The same report revealed the suboptimal performance of the health establishments concerned with healthcare service delivery because of their noncompliance with the prescribed quality assurance regulations. Furthermore, the continuous migration of quality assurance managers has been identified as being a factor contributing to noncompliance with quality standards, and poor-quality healthcare in South African public health establishments (Malakoane et al. 2020:3). Maphumulo and Bhengu (2019:5) reckon that the proposed plan to implement National Health Insurance (NHI) by the South African government requires adherence to quality standards in healthcare establishments, so as to realise their implementation.

Therefore, quality assurance managers are crucial for the supervision, execution and implementation of quality standards in health facilities (Mogakwe, Ally & Magobe 2019:4). Furthermore, the regulated norms and standards provide managers at all levels with information about the expected level of service delivery (NDoH 2013:23). In addition, Friebel et al. (2018:4) argue that quality assurance managers are critical to the continuous monitoring and improvement of healthcare service delivery processes. In the same manner, the Royal College of Nursing (2016:15) highlights that the responsibilities of quality assurance managers are the management of compliance, the implementation of organisational policies and procedures, and the provision of quality assurance in healthcare facilities. The same report finds that such responsibilities are often shared with the nursing managers involved, who should render efficient and effective patient care services. As a result, Maphumulo and Bhengu (2020:8) advocate the hiring of quality assurance managers to oversee the training and implementation of quality standards in all health establishments concerned. While the process of quality assurance is a continuous cycle requiring adherence in all healthcare establishments, it is a challenge for the quality nurse managers involved to implement the process in the absence of the support of all the stakeholders concerned, including the NDoH, the OHSC, the public–private partnerships, the sponsors from private companies and the customers or users of the services involved. According to Mogakwe et al. (2019:4), the quality assurance of healthcare delivery in the relevant institutions would likely improve if the health establishment management were to support the quality assurance implementation managers by developing standard operating procedures, training, quality improvement plans (QIPs) and providing support during clinic visits. Folkman, Tveit and Sverdrup (2019:102) highlight the importance of all managers working collaboratively together to optimise quality service delivery.

Although studies on quality standard adherence have been conducted, it was necessary to investigate quality assurance managers’ experiences with quality standard adherence in healthcare facilities because of the ongoing decline in healthcare delivery quality in South African public health facilities (Maphumulo & Bhengu 2019).

### Aim

The purpose of this study was to explore and describe the factors influencing quality standard compliance in health establishments in the Tshwane Municipality district based on the experiences of quality assurance managers so that supportive interventions could be suggested to improve compliance.

### Research question

What are the factors influencing quality standard compliance in health establishments in the Tshwane Municipality district?

### Definition of key concepts

#### Experience

Experience means the knowledge or skills that are gained through practical involvement or through exposure to a specific activity over a period of time (Oxford English Mini Dictionary 2017:199). In this study, the term ‘experience’ refers to the maintenance by quality assurance managers of their knowledge gained while implementing the prescribed quality standards.

#### Health establishment

A health establishment is a facility that provides patient treatment, therapeutic or diagnostic interventions, nursing or other health services (Republic of South Africa 2003:12). In this study, a health establishment refers to a public clinic or hospital in the City of Tshwane Metropolitan Municipality district in Gauteng province.

#### Quality assurance

Quality assurance is directly or indirectly related to performing activities conclusively, so as to ensure that they meet the desired quality of standards implementation and improved performance (Henker et al. 2018:180). In the present study, the term ‘quality assurance’ refers to the comparison of the healthcare quality provided to that which is required in the national standards, so as to enable the certifying of health establishments that meet such standards (South African Lancet National Commission 2019:77).

#### Quality assurance manager

The quality assurance manager is the manager who is in charge of overseeing and coordinating an organisation’s quality assurance process (Medicines Control Council 2017:5). In this study, the quality assurance manager refers to a person who is employed, or who is delegated, as a quality assurance manager in a public health establishment, such as a hospital, a primary health clinic or a community health centre, so as to oversee the provision of quality healthcare services.

#### Quality standards

Quality standards, according to Whittaker et al. (2011:60), are a set of principles that are used to evaluate establishments in relation to client expectations and needs. Quality standards are defined in this study as being the regulated norms and standards that apply to various categories of health establishments and were promulgated by the Minister of Health on 15 January 2018 (NDoH 2018:24).

## Research methods and design

### Design

A qualitative phenomenological design was used in this study for investigating the lived experiences of quality assurance managers who implement quality standards in public health establishments in the City of Tshwane Metropolitan Municipality. According to Neubauer, Witkop and Varpio (2019:91), a phenomenological design is appropriate for a researcher to use when wanting to explore participants’ lived experiences and the meanings associated therewith.

### Study setting

The setting was the public health facilities in the City of Tshwane Metropolitan Municipality district, Gauteng, South Africa, consisting of 14 hospitals, 8 community health centres and 65 primary healthcare centres in the municipality. As the municipality in the province had been found to underperform in terms of quality standards compliance (OHSC 2017:54), the area concerned was found to be the most appropriate setting for the study.

### Study population

The study population included all quality assurance managers working in health establishments and those assigned to district offices in the Gauteng province’s City of Tshwane Metropolitan Municipality. The study included only those quality assurance managers (operating in appointed and delegated positions) who had worked in their position for longer than a period of 6 months.

### Study sample

Non-probability purposive sampling was used to select the quality assurance managers who had worked in their position for longer than 6 months and who were willing to participate in the study. The researchers informed the (quality assurance) managers about the study, providing them with all the relevant information required. The participants who met the inclusion criteria were invited to participate in the study. The study’s purpose and benefits were explained to them, and any questions that they had to do with the study were answered. The researcher then scheduled the interview date, time and location for those who had agreed to take part in the research. The data collection began after receiving ethical clearance from the University of South Africa’s ethics committee, and permission from the Gauteng and the City of Tshwane Metropolitan Municipality district’s research committees. Nine quality assurance managers were interviewed in all, with the sample size being determined by means of data saturation. Data saturation occurs when the information that is obtained from the research participants produces no additional insights, but merely repeats information obtained from previously interviewed participants (Gray, Grove & Sutherland 2016:255; Saunders, Lewis & Thornhill 2019:315). After the seventh interview in the present instance, data saturation was reached, with the researchers concerned continuing with their interviews until the ninth participant was reached. The audio recordings were transcribed verbatim.

### Data collection

The required data, obtained between February 2021 and June 2021, were collected in the participants’ natural environment, consisting of the workplaces of the quality assurance managers involved, using an interview guide employing open-ended questions (Creswell & Creswell 2018:188). The researchers audio-recorded the interviews with the participants and took field notes to record such nonverbal cues as the facial expressions of the participants that were observed during the interviews, which lasted between 40 min and 60 min. As a result of the coronavirus disease 2019 (COVID-19) pandemic, the venues were prepared in advance in line with the prevailing COVID-19 protocol. While two participants were interviewed face-to-face, another two were interviewed using WhatsApp video calls, with the rest being interviewed through Microsoft Teams. According to Keen, Lomeli-Rodriguez and Joffe (2022:7), the challenges posed by COVID-19 in regard to the conducting of face-to-face interviews with the research participants involved could be addressed by means of collecting the required data by way of such virtual platforms as Microsoft Teams, Zoom and WhatsApp video calls. Each participant was asked: ‘Please share with me the factors influencing quality standard compliance in this health facility based on your experience as a quality assurance manager’. The request for information was followed by probes and prompts, when necessary, to obtain rich and detailed data from the participants concerned. By maintaining sound and respectful communication throughout, the researchers allowed the participants to express themselves freely. The researchers iteratively analysed the collected data, until the point of data saturation was reached.

### Data analysis

Each audio file collected during the interviews was transcribed within 2 days. The seven steps of Colaizzi’s thematic analysis framework for data analysis, as described by Polit and Beck (2017:540–541), were followed. Firstly, the researchers read all the transcripts concerned, so as to gain a thorough understanding of the experience of each quality assurance manager. Secondly, significant statements were extracted from the reviewing of each transcript. Thirdly, the meaning of each significant statement made was determined. Fourthly, the formulated meanings were organised into clusters of themes. Fifthly, the researchers integrated the results into a detailed representation of the experiences of the quality assurance managers, in relation to the implementation of quality standards. Sixthly, the representation of the experiences of quality assurance managers was clustered into subthemes, themes and superordinate themes. Lastly, a validation step was taken, in which some participants were contacted, in order to check that the analysed data obtained accurately portrayed their experiences and views and prevent any misinterpretation of the data. Furthermore, the researchers used the services of an experienced independent thematic coder who, after transcribing and analysing the collected data independently, came up with his own table of themes. The final table of themes, comprised two themes and subthemes, with relevant quotes from the participants’ interviews, was arrived at after comparing the researchers’ table of themes with the independent coder’s table.

### Trustworthiness

The strategies outlined by Lincoln and Guba (1985:296) as cited by Adekola and Mavhandu-Mudzusi (2021:5) were used to achieve trustworthiness, credibility, transferability, dependability and confirmability. The researcher established credibility by means of describing the study’s setting and participants. To ensure the proper capturing of the participants’ voices and nonverbal cues, the interviews were audiovisually recorded and transcribed verbatim. For validation, the services of an independent coder were used. To ensure transferability, the researchers described the research setting, the participants and the data collection method in detail, so that anyone who is interested in undertaking research into quality standards implementation has detailed information and can use the study’s findings in future research. The researchers highlighted the criteria used to sample the participants, as well as the key points from their demographic data. A comprehensive description and interpretation of the research method are provided to enhance the study’s dependability. The audit trail is described in terms of the keeping of all recorded information used during the data collection, the interview schedules, the data transcripts, the field notes and the notes about research procedures until the end of the study. Confirmability of the study was ensured by means of using verbatim quotations from the participants concerned. Furthermore, the researchers clarified the meaning of the nonverbal communication used by the participants, such as gestures and nodding of heads. The researchers used field notes, audio recordings and data transcripts to improve consistency, as well as follow-up interviews with some participants, so as to confirm the findings made.

### Ethical considerations

Before the data collection began, the researchers sought and received ethical clearance (ERC: HSHDC/1018/2020) from the University of South Africa’s Research Ethics Committee. Furthermore, the researchers obtained institutional approval from the Gauteng Provincial Health Department, the quality departments and the district municipalities concerned. Throughout the study, the following ethical standards were upheld: justice, beneficence, and respect for human dignity (Polit & Beck 2017:139–141). The participants were briefed on the study’s purpose, nature and potential impact on quality assurance implementation in healthcare facilities in the research setting. The researchers assured the participants that their participation was entirely voluntary and that they could opt out of the study at any time, without consequence to themselves. To ensure that their participation was voluntary, all nine quality assurance managers who took part in the study signed and returned their informed consent forms. The researchers protect the participants’ identities by means of using pseudonyms for them in the transcripts and when reporting the study’s findings. Furthermore, to prevent unauthorised access to the data concerned, the researchers vowed to keep the audiovisual files of the interviews, transcripts and field notes in password-protected electronic files.

## Findings of the study

### Biographical data

The participants’ ages ranged from 25 to 58 years. Of the participants, 78% (*n* = 7) were women. One-third (*n* = 3) of the participants had a qualification in quality assurance, while one participant was studying towards a qualification in the Quality discipline at the time of the study. All of the participants were appointed as quality assurance managers. Four participants were from different hospitals and each had worked in more than two facilities doing quality assurance. One was from a community health centre and worked as a quality assurance specialist at more than two facilities. The remaining four were from primary healthcare facilities in the district. The data analysis of the interview transcripts revealed two themes: (1) the motivators of quality standard adherence and (2) challenges to the implementing of quality standards.

### Theme 1: Motivators of quality standard adherence

The study’s findings revealed that the legislative requirements and the national health regulations were the motivators of adherence among the quality standard managers.

#### Legislative requirements

The study showed that legislative requirements such as regulated norms and standards tend to promote adherence to quality standards among the study participants.

#### Regulated norms and standards

The findings indicate that the quality assurance managers fostered compliance with the quality standards as required by law. The participants revealed that the quality assurance managers’ legal obligations to adhere to the quality assurance guidelines promoted the health establishments’ compliance with the regulated norms and standards:

‘I had to explain that Regulated Norms and Standards is a legislation that we should comply with, and, at some stage, I had to seek assistance of other stakeholders, so that we sing in one voice.’ (P2, female, 51 years old)

‘Some of the nurses were rude back then. Patients did not know their right[*s*] back then. Now, there are legal frameworks and regulations that we need to abide by, such as the Patients’ Rights Charter, Batho Pele principles, constitution of the country.’ (P4, female, 45 years old)

The participants also noticed that the legislative climate created by the implementation of the regulated norms and standards improved the health establishments’ adherence to quality assurance standards:

‘The main aim for this [*sic*] Norms and Standards is to bring improvement in the facilities, of which we can’t deny [*there is a*] big improvement in our healthcare facilities. So, there is progress, as we see reduction in number of errors, meaning that there is improvement in patient satisfaction.’ (P1, female, 40 years old)

In addition to the norms and standard regulations, the institutions involved were expected to adhere to quality standards, as prescribed by the national health regulations.

#### National health regulations

The need to comply with national health regulations was a motivating factor for adhering to the quality assurance standards in the study setting. The study’s findings showed that the two components of the national health regulations, namely the ideal Clinic Realisation programme and the Ideal Hospital Realisation programme, enhanced adherence in the health establishments in the research setting:

‘When National Core Standard and Ideal Clinic Realization programme were introduced, it changed the mindset for the nurses, we now know that we don’t just go to work just to see patients. We need to take certain things into considerations. We need to respect the patient’s privacy. As the managers, you don’t just go to work and sit in the office, because there is a lot of things you need to do. Things have changed a lot.’ (P3, male, 28 years old)

The implementation of national health regulations in health establishments was, at the time of this study, monitored by the OHSC. The participants alluded to the guidelines provided by the regulations as motivating compliance with the quality assurance standards in the health facilities concerned:

‘We conduct the self-assessments, ideal hospital assessments, clinical audit, patient safety incidents and patients give us feedback about the service they received. We draw [*up*] quality improvement plans (QIPs) and ensure that the gaps are closed, in order for our institution to comply with the Regulated Norms and Standards that are monitored by the Office of Health Standards Compliance.’ (P1, female, 40 years old)

Besides mentioning the motivators of adherence to the quality assurance standards in place, the study also revealed the challenges that the quality assurance managers faced while trying to implement the quality assurance standards in health establishments in the research setting.

### Theme 2: Challenges in implementing quality standards

The study’s findings revealed that the human and material resources and the inadequate infrastructure were barriers to the implementation of quality assurance in the health facilities located in the research setting.

#### Human resources

The study’s findings highlighted various human resource challenges in the health establishments that hindered the implementation of quality assurance in the study setting. The challenges included a shortage of personnel, staff attitudes, inadequately trained staff and the improper placement of staff.

#### Shortage of staff

According to the study findings, one of the challenges impeding proper implementation of quality standards was staff shortage:

‘The biggest challenge is staff shortage, because it is very difficult to deliver quality patient care when you are short-staffed. People are overworked.’ (P1, female, 40 years old)

‘You can have an appointment with the manager in the ward to do QIP, but when you reach the ward, you find that she is busy with patient care, due to staff shortage, and she is unable to do admin duties.’ (P2, female, 51 years old)

#### Staff attitudes

The results of the study indicated that staff attitude was a major determinant of the quality of service rendered in a healthcare facility. The participants reported encountering negative attitudes from their co-workers, while implementing quality standards:

‘Monitoring of waiting times is one of the requirements, but the professional nurses are supposed to fill in the time that the patient spent in the consulting rooms, but they ignore and leave it blank, and, when you talk about it, they will tell you that they are nursing the patients and pushing the queue, rather than nursing the paper.’ (P5, male, 37 years old)

A participant reflected that the patients involved were subjected to negative attitudes from the nurses at the health facilities involved, in addition to such attitudes being directed at the quality assurance managers themselves:

‘I don’t know if it is because of the COVID or what, but the nurses’ attitude is bad; not all the nurses, though. There are those who treat patients like they are doing patients favours.’ (P9, female, 58 years old)

#### Inadequate training of staff

A lack of staff training was found to be a factor affecting the implementation of quality standards in the health establishments in the research setting:

‘I don’t have any … formal training [*in*] quality management. I have been requesting that my employer assist, but my request is not considered. To them, it’s okay. As long [*as*] I am doing the job okay, it’s fine. There are courses in quality, but they are expensive, and I can’t afford that on my own.’ (P5, male, 37 years old)

‘So, we leave these employees that are supposed to help us so much in the implementation of quality standards without capacitating them. Not that they don’t want to come to the party, but we are not doing enough to capacitate them.’ (P3, male, 28 years old)

#### Improper placement of staff

Another factor that was cited by the participants as impeding the implementation of quality standards was the improper placement of quality assurance staff:

‘Quality directorate in our facilities struggle to retain staff. Remuneration is low, because it is not a specialising department. Quality managers in the hospitals get … different pay from the nurses, but with us [*it*] is different. Now, some employees don’t take me serious[*ly*], because they earn more than me, and I am a junior nurse with no qualification in the field of quality.’ (P5, male, 37 years old)

Besides the human resources involved, the study showed that the lack of material resources also hindered the implementation of quality standards in the health establishments located in the research area.

### Material resources

The research findings made highlighted the shortage of materials, the ineffective procurement processes, and the mismatch between policies and resources as the material resources-related factors that challenged the implementation of quality standards in the health establishments in the research setting.

#### Shortage of materials

The findings revealed a scarcity of the working materials required daily to provide basic patient care, including the critical equipment used to provide emergency services in the health establishments concerned:

‘When we order some items needed for the emergency trolley, they are not available, [*and*] there is no stock. We order certain items many times, and we are told that they are out of stock, and it end[*s*] there, with just saying it is out of stock.’ (P5, male, 37 years old)

#### Ineffective procurement processes

Participants also identified the ineffective procurement processes as a barrier to the effective implementation of quality standards in the research setting:

‘This tendering issue is just an impediment. Sometimes, we receive replacement parts that are not compatible with the equipment at hand. The problem is with the supply chain processors, who have no background of health, yet they do not take recommendations from the users of equipment. It is something that is causing all the chaos − prices get overinflated.’ (P4, female, 45 years old)

#### Mismatch between the policies and the resources

The implementers of the policies on quality standards were found not to be adequately involved in the budgeting and spending processes, with their requests for monetary funds required for the optimum implementation of quality standards being overlooked. While the government was said to intend to raise the levels of quality standards in all healthcare facilities, the findings revealed fragmentation in the implementation processes because of misalignments between the government policies and the available resources:

‘The problem is that we have the best policies [*and*] we have the budget, but, somewhere, there is miscommunication between the two. Where the standard says for a facility to be considered ideal, the facility should have A, B, C and D. We then give facility a checklist to say, ‘Check the following aspects’. However, we don’t give them [*a*] budget to fix the things that should make that clinic ideal, so the policy itself is very great, but [*it doesn’t prevent*] ineffective management. I don’t know if the management of finance or its politics is, but if they were coming to the party, we would have, by far, the best facilities ever, but, right now, there is mismatch between the implementation of policies and available funds.’ (P3, male, 28 years old)

Apart from the material resource constraints, the dilapidated and inadequate infrastructures in place were found to hinder the implementation of quality standards in the research setting’s health establishments.

### Infrastructure-related barriers

The participants observed that the dilapidated infrastructures in the health facilities concerned contributed to the noncompliance with quality standards, as outlined in the following quotation:

‘The building should be in good condition; at the moment, the structure of a building is very old, and [*it*] is not in good condition, and it does not suit to be a health establishment. We are working in this building, because we do not have an alternative.’ (P5, male, 37 years old)

The participants reported that a lack of adequate infrastructure throughout all health establishments in the research setting was a barrier to the meeting of the set quality standards:

‘Our facility is very small, and we must render all the comprehensive services. It is really tough, especially when it comes to social distancing and isolation rooms. If they can build a bigger clinic, that would really help a lot, because now, you find that patients are delivering in [*the*] admission area, because the delivery room is full, and the toilets are insufficient, just to name a few.’ (P8, female, 27 years old)

‘It is unacceptable that the facility can run with one or two computers. I have written many motivation letters, but is [*i.e. they are*] not helping. The best assistance that I can do is to borrow equipment from the facilities nearby. However, you find that they are experiencing the same challenge. The computers and other equipment, such as [*the*] defibrillator, need to be procured; my hands are tight.’ (P6, female, 44 years old)

## Discussion

The study’s findings revealed that the employees in healthcare facilities in the research setting were motivated to meet quality standards because they felt obligated to abide by both the legislative requirements and the national health regulations. The findings made concurred with those of the World Health Organization (WHO 2018:34), which states that healthcare practitioners in a health establishment have an inherent accountability and responsibility to ensure that the healthcare services provided meet the expected quality standards. In the same vein, such motivation aligns with the expectations set in the national core standards (NDoH 2021:2). While the managers involved were tasked with being the gatekeepers of quality healthcare delivery (Maphumulo & Bhengu 2020:12), the national health regulations guided the managers and staff on the quality tools used in evaluating the quality standards, paving the way for the implementation of the NHI policy. However, Maphumulo and Bhengu (2019:5) caution that policies and regulations alone are insufficient to ensure the adherence to quality standards in healthcare service delivery; rather, such regulations must be implemented and followed through on.

Aside from revealing the motivation for quality standard compliance, the findings show that such human resource factors as a shortage of personnel, negative staff attitudes, inadequately trained staff and the improper placement of staff are all obstacles to quality standard implementation and compliance in health establishments. Consequently, due to a shortage of nursing staff in the health establishments concerned, the quality assurance managers have had to augment the number of staff personnel in the clinical departments, thus postponing their quality monitoring roles. Doing so has had negative effects on the continuous monitoring of quality healthcare standards in health establishments. The attitude of personnel has also played a role in implementing the quality standards concerned. These findings are consistent with those made by Lephogole (2018:39), who cites that the staff shortage has negatively affected the delivery of quality healthcare and the proper implementation of the set quality standards. Although the population has increased for several different reasons, without a corresponding increase occurring in the number of healthcare staff, the situation has placed a strain on those health facility personnel scheduled to serve a wide range of patients. Mutshatshi et al. (2018:3) argue that the concomitant staff shortage has compromised the quality of the standards set for the services rendered. Likewise, Fryatt and Hunter (2015:28) report that, if the standardisation of the staff–patient ratio is not addressed in the relevant health establishments, it will be difficult to control the personnel shortages and to ensure the maintenance of quality standards.

Furthermore, the results obtained indicate that the quality assurance managers encountered negative attitudes from the staff regarding the implementation of quality standards. They also considered quality managers as patients’ lawyers, who only visited the health facilities concerned when there were patient complaints. Such findings corroborate Khambani, Mugova and Cebekhulu’s (2017:954) findings, which established that the employees might display negative attitudes towards quality improvement efforts, due to their resistance to change and owing to the lack of discipline. Similarly, this study’s findings agree with those of Kumbi et al. (2020:7), who report that the negative attitude of healthcare workers towards their managers could impede the implementation of quality standards. The study’s findings showed that the poor staff attitude displayed at the time of the present research was not only limited to the quality assurance managers but also towards the patients involved. These findings correspond with those of Khoshakhlagh et al. (2019:9), who observed that the poor attitudes of the staff towards their patients negatively affected the provision of quality care in health establishments. The authors suggest that the poor staff attitude could have been because of a lack of motivation, poor training and the lack of resources, among others. Haskins, Phakathi and Horwood (2014:32) observe that the negative attitude of the personnel causes a deterioration in the quality of patient care. Therefore, quality assurance managers should constantly monitor the attitudes of staff, so as to prevent poor healthcare service delivery to the patients involved.

Apart from the negative staff attitudes, the findings of study indicate that the inadequate training of staff also posed a challenge to the implementation of quality assurance in health establishments in the research setting. The findings elucidate the difficulties experienced by the quality assurance managers involved, who were not suitably trained to execute their responsibilities in such a capacity. The data analysis showed that those nurses who were tasked with quality management were supposed to pay to attend the requisite courses themselves, which most of them could not afford to do. Such findings are in line with those made by Serrate (2019:4), who indicates the significance of strengthening the delivery of quality standards through continuous training and education.

This study further revealed that the improper placement of quality assurance managers impeded the implementation of quality standards. Furthermore, this study indicates that those who were responsible for quality assurance, at the time of the research, were not formally appointed to their positions. Therefore, they were often distanced from the responsibilities that they had to fulfil, based on their facility’s needs. Such findings were supported by Kakyo and Xiao (2017:248), who documented that, when there was a shortage of personnel in the health establishments surveyed, the quality assurance personnel tended to be delegated to perform other clinical duties.

The results showed that besides the numerous human resource challenges, material resource-related factors such as the shortage of materials, the ineffective procurement processes, and the mismatch between policies and resources, contributed to the poor implementation of quality standards in the health establishments concerned. The findings agreed with those made by Maphumulo and Bhengu (2019:2), who stated that the shortage of equipment and supplies was among the major causes of noncompliance with the quality standards in place. Likewise, Manyisa and Aswegen (2017:37) argued that a shortage of critical healthcare materials and equipment tends to undermine the ability and willingness of the quality assurance managers and staff to comply with the quality standards that might otherwise be capable of being maintained at the health facilities in question.

The results obtained also identified ineffective procurement processes as being another factor hindering proper quality standard implementation. While the NDoH (2017:17) has acknowledged that it has weak control and monitoring processes over procurement, leading to poor service delivery in the public health sector, Hunter et al. (2017:121–122) call for both the national and the provincial health departments to correct the procurement processes that have led to many health establishments functioning without the required medication, consumables, equipment and furniture. In line with the findings of this study, several studies allude to the chronic underfunding of health establishments as being a major barrier to the effective implementation of quality standards in health establishments (Malakoane et al. 2020:2; Modisakeng et al. 2020:6).

Furthermore, this study found that the inadequate infrastructure contributes to the poor compliance attained with quality standards in research health facilities. Both staff and patients were discovered to have been exposed to a decrepit infrastructure that was unfit for providing a high standard of healthcare. The findings also show the lack of a sound infrastructure for accommodating patients who jeopardised their privacy and diminished their dignity, while they were seeking healthcare. Patients were made to wait outside the concerned healthcare facility, so that the personnel could perform tasks critical to infection prevention and control, as well as to basic essential healthcare. The inadequate infrastructure was found to impede the effective implementation of quality standards. As a result, the need for ideal health establishments was seen as being critical. The findings obtained agree with those of Mogakwe, Ally and Magobe (2020:6, 11–12) and Manyisa and Aswegen (2017:35), who explain that a faulty infrastructure and a lack of space for conducting various programmes in primary healthcare facilities tend to compromise patient privacy, impede service delivery and impact care quality negatively.

### Limitations of the study

The present study was limited to one of Gauteng province’s municipal districts, namely the City of Tshwane Metropolitan Municipality district. Included in the study were only nine quality assurance managers, who had been appointed or delegated to work as quality assurance managers and quality assurance champions and who had been in their position for over 6 months, declared that they were willing to participate in the study. The findings of this study were based on the experiences and perspectives of the selected quality assurance managers, which implies that the views of other stakeholders were missing from this study. While the study’s findings provided an in-depth understanding of the issue under investigation, it should be interpreted with the above-mentioned limitations in mind.

### Recommendations

The barriers to compliance and the implementation of quality assurance identified in this study should be addressed, so as to improve compliance with the quality standards set for the health establishments in the City of Tshwane Metropolitan Municipality. Adequate resources should be allocated to the health sector by all levels of the government, so as to address the human resource constraints, as well as the material and infrastructural challenges that are impeding the effective implementation of, and compliance with, the quality standards set for the health establishments located in research settings. While the barriers concerned are being addressed, the management of the healthcare facilities should use the motivators of quality standards compliance to promote long-term acceptance of their implementation.

## Conclusion

This study explored and described the lived experiences of quality assurance managers regarding the implementation of quality standards at public health facilities in the City of Tshwane Metropolitan Municipality district, South Africa. The findings of the study revealed the motivators and challenges to the implementation of quality standards in the research setting. It was noticed that the National Core Standards and other regulations and policies have paradoxical roles in compliance with the quality standards in the health establishments in the study setting. Based on the findings of the study, the identified challenges to compliance should be addressed, and the identified motivators should be leveraged, so as to promote the maintenance of quality standards in healthcare delivery services in the research setting. The study established the existence of a shortage of quality assurance personnel and support in the implementation of quality standards in the public health establishments in the City of Tshwane Metropolitan Municipality. The study’s findings could influence the allocation of needed resources by DOH for recruitment of human resources and training programmes for the quality assurance personnel involved, so as to optimise the implementation of quality standards in the health establishments in the City of Tshwane Metropolitan Municipality district and in similar settings.
